# Circadian rhythms in metabolism and mental health: a reciprocal regulatory network with implications for metabolic and neuropsychiatric disorders

**DOI:** 10.1016/j.cophys.2025.100836

**Published:** 2025-09

**Authors:** Matthew Lloyd, Brooke A Prakash, Lucy Zhao, Guohao Ni, Yining Ru, Sridhar R Vasudevan

**Affiliations:** Department of Pharmacology, University of Oxford, Mansfield Road, Oxford OX1 3QT, UK

## Abstract

Circadian rhythms orchestrate metabolism and brain function, aligning internal physiological processes with the 24-hour day–night cycle. Growing evidence highlights a reciprocal relationship between circadian regulation, metabolism, and neurobiological processes. Circadian disruption impairs glucose and lipid homeostasis, alters neurotransmitter and endocrine signalling, and triggers stress response, forming a feedback loop that impacts metabolism and brain function. These disturbances are implicated in many conditions, such as obesity, diabetes, depression, and bipolar disorder. This review examines recent advances in the interplay between circadian regulation, metabolism, and mental health, emphasising shared molecular mechanisms and their role in disease progression. Understanding these connections may ultimately inform therapeutic strategies that integrate circadian-based approaches to improve treatments for metabolic and psychiatric disorders.

**Current Opinion in Physiology** 2025, **45**:100836

This review comes from a themed issue on **Circadian Rhythm**

Edited by **Martin Young, John O'Neill** and **Linda van Laake**

For complete overview of the section, please refer to the article collection, “Circadian Rhythm (2024)”

https://doi.org/10.1016/j.cophys.2025.100836

2468–8673/© 2025 The Authors. Published by Elsevier Ltd. This is an open access article under the CC BY license (http://creativecommons.org/licenses/by/4.0/).

## Introduction

**Figure 1:**
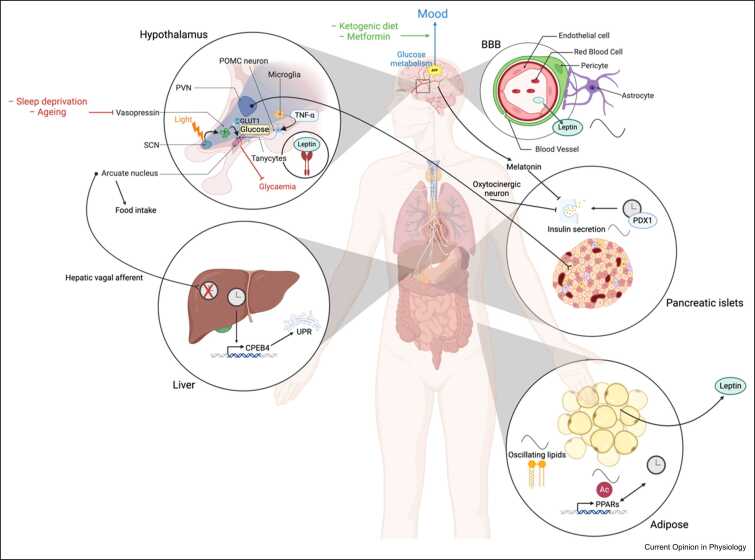
An overview of interconnected molecular mechanisms underlying circadian, metabolic, and psychiatric dysfunction, focusing on the brain, liver, adipose, and pancreas, and the crosstalk between these organs. Created in BioRender.

Circadian clocks are endogenous 24-hour oscillators coordinating physiology and behaviour to the appropriate time of day. The mammalian circadian clock functions through an autoregulatory feedback loop driven by transcription and translation [1]. The suprachiasmatic nucleus (SCN), located in the anterior hypothalamus, serves as the master circadian clock. It is entrained by external light–dark cycles. Working with peripheral clocks, the SCN regulates a wide range of physiological functions. These include synchronising metabolic, endocrine, and neural processes to optimise energy balance, cognitive function, and emotional stability^2^, ^3^, ^4^ (Figure 1). These rhythms are essential for maintaining metabolic homeostasis and brain function via their influence on neurotransmitter signalling, stress adaptation, and neurobehavioral function.

Disruptions in circadian timing — whether due to genetic mutations, sleep deprivation, or shift work — are strongly associated with metabolic disorders, such as obesity, insulin resistance, and type 2 diabetes (T2D)^5^, ^6^, as well as psychiatric conditions, including major depressive disorder (MDD), bipolar disorder (BD), and anxiety 7, 8. The bidirectional link between metabolism and neuropsychiatric function is coordinated by various mediators. These include insulin, leptin, glucocorticoids, and inflammatory pathways, which regulate energy utilisation and neurotransmitter release.

The brain-periphery dialogue is maintained by hypothalamic circuits, the autonomic nervous system, and hormones and immune signalling, ensuring that metabolic rhythms align with behavioural and emotional states. The connection between metabolism and mood is exemplified by longitudinal studies in patients with BD, who exhibit significant sleep–wake abnormalities and receive treatment for diabetes and hyperlipidaemia at a younger age compared to the general population [9]. Conversely, diabetes is associated with markedly worse outcomes for mood symptoms, a finding reinforced by extensive preclinical and cohort studies [10].

At a mechanistic level, several genes such as *CLOCK, BMAL1*, and *PERIOD*, which are intimately involved in the generation and regulation of circadian rhythms and sleep, have been linked to psychiatric and metabolic disorders in humans 11, 12, 13 and supported by mouse models. *CLOCK* mutant mice [14], for example, not only display sleep and circadian deficits, but also show reduced anxiety and depression-like behaviours, increased risk-taking, and substance consumption consistent with mania [14], alongside severe metabolic abnormalities 5, 15. This is indeed observed with other circadian gene mutants; we refer to comprehensive reviews that explore the impacts of sleep and circadian disruption — whether induced genetically, through environmental manipulations in mice, or socially or experimentally in adolescents — demonstrating increased risk of cardiometabolic and neuropsychiatric disorders. 13, 16, 17, 18, 19.

Clinical evidence further supports the role of circadian disruptions in these disorders, and addressing these disorders can improve both mood and metabolic health. Chronotherapies, such as acute sleep disruption and bright light therapy that reset circadian gene expression rhythms^16^, ^20^, have been shown to improve depressive symptoms in up to 60% of human patients, even in those who are drug-resistant. Similarly, timing food intake to the correct biological time (day) in night shift workers has been shown to alleviate circadian desynchrony and improve glucose tolerance and mood in these individuals^7^, ^8^.

This review examines the complex interplay between the circadian clock, metabolism, and mental health, highlighting recent research on key regulatory mechanisms across cellular, organ-specific, and systemic levels (Figure 1). While previous studies have explored the role of sleep and circadian regulation of metabolism and psychiatric disorders, we focus on their shared mechanisms and emerging bidirectional influence, which is increasingly recognised as a critical factor in the pathophysiology of both metabolic and psychiatric conditions 21, 22. Specifically, we emphasise the roles of (1) glucose homeostasis, (2) lipid signalling, and (3) cellular stress in connecting circadian rhythms, mood, and metabolic regulation. By framing these interactions within a broader physiological and pathological context, we provide insights into how circadian, metabolic, and mental disorders mutually influence one another and how restoring circadian integrity may offer therapeutic benefits for both metabolic and neuropsychiatric health.

## The bidirectional link between glucose homeostasis and psychiatric disorders

Understanding how circadian rhythms regulate glucose metabolism offers an illuminating entry point into the broader intersection of metabolic and mental health. We begin by examining how disruptions in glucose homeostasis influence and are influenced by psychiatric states. Brain-periphery crosstalk is essential for coordinating nutrient intake and metabolism across organ systems with correct diurnal timing. Emerging evidence suggests that peripheral metabolic signals, including hormones, cytokines, and gut-derived metabolites, are regulated by the circadian clock and play a crucial role in regulating mood and emotional states. Consequently, disruption of the circadian clock not only impairs glucose homeostasis, leading to the development of metabolic syndrome 23, 24 but may also contribute to the observed links between metabolic dysfunction and affective disorders due to the loss of proper brain-periphery communication and alterations to the brain’s energy sources[21].

### Neuropeptidergic regulation of glucose homeostasis

The SCN orchestrates these diverse physiological effects through the release of crucial neuropeptides, such as vasopressin. These serve as vital mediators of clock-controlled metabolic and behavioural rhythms. Suprachiasmatic vasopressin synthesis follows a robust circadian rhythm and influences multiple cardiometabolic physiologies 25, 26. Recent data also demonstrate that vasopressin from SCN projections stimulates GLUT1 expression in tanycytes. This barrier-forming macroglial cell lines the third ventricle and modulates hypothalamic neuronal activity to regulate energy balance [27]. Vasopressin-induced GLUT1 expression promotes glucose entry into the arcuate nucleus, thereby lowering peripheral glycemia [28]. High tanycytic GLUT1 expression coincides temporally with the transition from activity to rest, enabling increased arcuate glucose uptake in anticipation of a period of lower metabolic demand.

Disruptions to sleep and circadian rhythms can have notable effects on metabolism via this pathway. For example, sleep loss decreases suprachiasmatic vasopressin activity, causing hyperglycaemia by reducing arcuate glucose entry via tanycytes [29]. Vasopressin also declines with age, mirroring the decline in circadian rhythm robustness seen in ageing and T2D 30, 31. This suggests a persuasive mechanism by which circadian rhythms can regulate glucose homeostasis and how their disruption contributes to the development of metabolic disorders.

### Rhythmic brain barrier function in energy homeostasis

In addition to regulating glucose entry, the circadian clock also influences other brain functions, such as the integrity of brain barriers, which are essential for maintaining central metabolic and mental health. The loss of blood–brain barrier (BBB) integrity is implicated in psychiatric disorders, including schizophrenia and mood disorders such as MDD and BD [32]. The BBB is comprised of vascular endothelial cells connected by tight junctions and surrounded by pericytes and astrocyte end feet, which regulate their function. Most molecules cross the BBB transcellularly, allowing for control over transport between the brain and the periphery [33]. An endogenous BBB circadian rhythm results in oscillating permeability to hormones, metabolites, and drugs. This is due to variations in key transporter activity [34], likely driven by oscillations in intracellular magnesium as a cofactor for ABC transporters [35].

Tanycytes, beyond their role in glucose transport, play a crucial role in leptin uptake alongside the BBB. Ablation of the tanycytic leptin shuttle (selective LepR deletion) causes impaired insulin secretion, accompanied by downregulation of key identity genes and upregulation of unfolded protein response (UPR) markers, indicating cellular stress in pancreatic β-cells [36]. Since both circulating leptin levels and BBB permeability exhibit circadian fluctuations, brain leptin uptake displays a corresponding diurnal variation, peaking during the active phase (ZT14-ZT18 in mice), potentially leading to disparities in leptin concentrations between the brain and the periphery [37]. The saturable nature of BBB leptin transport could mean that mistimed feeding reduces the proportion of circulating leptin reaching the brain, attenuating satiety signals and potentially contributing to hyperphagia and hyperglycaemia.

### Neuroendocrine and circadian control of pancreatic insulin secretion

Beyond central regulation, circadian influences on peripheral organs, such as the pancreas, are equally critical in shaping metabolic rhythms. We next examine how neuroendocrine circuits and intrinsic pancreatic clocks synchronise insulin secretion with daily physiological demands. Peripheral glucose homeostasis relies on pancreatic islet cells, balanced insulin, and glucagon secretion. Sympathetic activation stimulates glucagon release and inhibits insulin secretion via β₂- and α₂-adrenergic receptors, while parasympathetic activity promotes insulin release [38]. Douglass et al. found that microglial Tumor Necrosis Factor-α, an agent capable of modifying SCN function and also exhibits a robust diurnal rhythm, stimulates POMC neurons in the arcuate nucleus, leading to parasympathetic-driven amplification of first-phase insulin secretion and improved glucose tolerance [39]. However, this pro-inflammatory activation is independently associated with hyperphagia and adiposity, revealing a trade-off between glucose homeostasis and weight regulation.

Neurotransmitter signalling also directly regulates insulin secretion by modulating insulin granule docking — a key rate-limiting step in secretion 40, 41••. Papazoglou et al. [41] identified a novel circadian mechanism whereby oxytocinergic PVN neurons directly regulate β-cell function, suppressing insulin secretion to increase glycaemia. This pathway may indeed contribute to the synchronised rhythmicity of basal insulin secretion. Furthermore, in PVN BMAL1 knockout models, disrupted oxytocin rhythms altered peripheral gene expression, energy intake, and metabolism [42]. Additionally, the pineal gland reinforces circadian regulation of β-cell activity via melatonin, which reduces insulin secretion by lowering cAMP levels. The MTNR1B gain-of-function variant (rs10830963) identified as a risk factor in genome-wide association studies enhances β-cell melatonin receptor expression, correlating with elevated fasting glucose and increased T2D risk [43].

Beyond neural circadian regulation of pancreatic insulin release, β-cells are also under endogenous circadian regulation at the transcriptional level. PDX1 binds with CLOCK/BMAL1 at enhancers to induce rhythmic transcription of genes linked to insulin secretion [44]. Further complexity in brain-periphery crosstalk arises from signals of liver dysfunction relayed to the arcuate nucleus via the vagus nerve. Hepatocyte-specific deletion of REV-ERBs causes hyperphagia and mistimed feeding, disrupting the arcuate transcriptome, which is prevented by selective ablation of the hepatic vagal afferent nerve [45]. Collectively, these lines of evidence demonstrate the numerous ways in which circadian rhythms regulate glucose and energy homeostasis.

### Role of circadian glucose regulation in neuropsychiatric function

Given that glucose is the primary energy source for the brain, it is perhaps unsurprising that dysregulation in cerebral glucose homeostasis and vasopressin signalling, key regulators of brain energy status, are implicated in severe mental illness (SMI) with regional variations in cerebral glucose metabolic rate correlating with mood states 46, 47. Daytime eating in shift workers mitigates both glucose intolerance and mood disturbances, suggesting a link between metabolic regulation and mental health 7, 8. These findings have led to a recently developed hypothesis proposing that manic states involve astrocytic hyperactivity driving increased glycolysis, glutamine synthesis, and subsequent neuronal glutamate production, resulting in elevated energy metabolism [21]. Supporting this, increased hippocampal glutamate is observed in BD patients with higher BMI [48].

In parallel, there is growing evidence that insulin sensitisation leads to mood stabilisation. Since insulin signalling and glucose regulation are under circadian control, improved circadian function may contribute to this effect. Supporting this, a recent randomised controlled trial evaluating metformin, a first-line antihyperglycemic agent, significantly improved the depression rating scores in treatment-resistant BD patients. Participants who resolved insulin resistance showed sustained improvements in depression symptoms throughout the entire 26-week trial [10]. Several mechanisms may explain these findings. Metformin upregulates BMAL1 [49]. Insulin and Insulin-like Growth Factor-1 signalling synchronise local clocks throughout the body, and mistimed insulin administration disrupts circadian rhythms [50]. Additionally, insulin and glucose have a direct impact on brain function. Together, these findings point to a feedback loop where circadian dysfunction exacerbates metabolic issues while metabolic disturbances further impair circadian clock function, particularly in tissues with high metabolic demand, such as the brain.

These insights reinforce the notion that therapeutic interventions targeting metabolic regulation may benefit circadian stability, potentially contributing to improved cognitive and mood outcomes in metabolic and neuropsychiatric disorders. Reflecting this relationship, it is not surprising that ketogenic diets (which shift brain energy supply from glucose), anti-diabetic drugs, and vasopressin modulators are under investigation as potential treatments for psychiatric disorders 46, 51•, 52•.

### Rhythmic lipid signalling as a metabolic and psychiatric regulator

While glucose metabolism has received significant attention, circadian control of lipid signalling is increasingly recognised as a parallel mechanism linking metabolic dysfunction to mood disorders. We now explore how rhythmic lipid pathways and their nuclear receptors influence systemic and brain health, maintaining a bidirectional relationship with the circadian clock. Alongside high blood glucose, impaired lipid homeostasis is a hallmark of metabolic syndrome, characterised by elevated serum triglycerides, low High Density Lipoprotein-Cholesterol levels, and excessive visceral fat accumulation. Numerous studies have demonstrated that circadian disruption contributes to the development of metabolic syndrome 5, 53, 54. Similarly, lipidomic rhythmicity is altered in T2D, with lipids exhibiting a phase desynchrony between serum and subcutaneous adipose [55]. Circadian rhythms in fibroblasts from T2D patients correlate with patient Haemoglobin A1c levels [56]. Similarly, fibroblasts from BD patients display circadian abnormalities [57]. Though the mechanism relating Haemoglobin A1c levels to fibroblast rhythms in T2D remains incompletely understood and it is uncertain whether circadian dysfunction in BD fibroblasts correlates with metabolic abnormalities, these findings suggest a plausible link between cellular circadian dysregulation and the metabolic disturbances characteristic of both T2D and BD.

Peroxisome proliferator-activated receptor (PPAR) transcription factors, acting as lipid-responsive receptors, are key mediators of crosstalk between the circadian and metabolic systems. They regulate both lipid and carbohydrate metabolism. CLOCK/BMAL1 induces PPAR-α, and in turn, PPAR-α and PPAR-γ, along with PGC-1*α,* activate BMAL1 [58]. PPAR-γ is the isoform most expressed in adipose tissue, being essential for promoting adipocyte differentiation and lipid storage. In addition to demonstrating mRNA- and protein-level circadian rhythms, PPAR-γ exhibits a daily acetylation rhythm (with a peak at ZT0 and a trough at ZT18) [59]. In obesity, PPAR-γ expression in white adipose tissue decreases and loses its rhythmicity, which may indirectly lead to the downregulation of BMAL1. In line with this concept, the PPAR-γ agonist pioglitazone prevents the disruption of clock gene rhythmicity in the livers of mice on a reverse (daytime) feeding protocol [60]. PPAR-γ agonists are routinely used clinically against T2D, acting on multiple insulin signalling pathway sites [58].

PPAR-γ agonism is also explored for treating SMI, such as BD and MDD. Indeed, clinical studies using PPAR-γ agonists to target mood have reported improved patient outcomes 61, 62. Although the precise mechanism remains under investigation, current lines of evidence suggest that the therapeutic benefits may arise from reduced neuroinflammation and elevated levels of neurotrophic factors, including neurotrophic factor-α1 and brain-derived neurotrophic factor, as well as enhanced neurogenesis [63]. Moreover, emerging research indicates that PPAR signalling can be activated by a ketogenic diet, which is gaining attention as a potential treatment for neuropsychiatric disorders [52]. While several explanations have been proposed for the mechanisms of the ketogenic diet, an attractive possibility is that the ketogenic diet increases rhythmic PPAR-γ activity and boosts circulating ketone bodies 64, 65, though causality remains to be established. This raises the intriguing possibility that PPARs play a role in the mood-regulating effects of ketogenic diets alongside their effects on neuroinflammation and neurogenesis, all of which are independently being considered as viable drug targets.

### Cellular stress responses in sleep and circadian biology: beyond the hypothalamic-pituitary-adrenal axis

It is well established that sleep disturbances and circadian misalignment influence metabolic 19, 66, 67, 68 and psychiatric function 22, 69, with the hypothalamic-pituitary-adrenal axis playing a role [70]. However, recent investigations have illuminated additional stress pathways, such as endoplasmic reticulum (ER) stress and oxidative stress, as independent contributors to disease pathophysiology. This section explores how these disruptions regulate key cellular stress pathways, further clarifying the metabolism-psychiatric connection.

### Circadian regulation of cellular stress pathways

Circadian clocks regulate cellular stress responses to align them with the demands of nutrient use and protein folding. In endocrine tissues with high secretory load, UPR components, such as binding immunoglobulin protein (BiP), exhibit circadian rhythmicity [71]. Disruption of these rhythms impairs function, as seen in mouse SCN and fibroblasts, where eIF2α kinase 4 modulates Per2 transcription via ATF4 [72]. Similarly, ER-stressed fibroblasts exhibit reduced circadian gene expression, while BiP overexpression or chemical chaperone treatment restores rhythmicity [71].

The UPR–circadian interaction is exemplified by cytoplasmic polyadenylation element–binding protein 4 (CPEB4), which governs pre-mRNA processing and translation of mRNAs containing cytoplasmic polyadenylation elements. Its hepatic transcription is clock controlled, and translation is activated in response to ER stress. Liver-specific CPEB4 deficiency leads to steatosis and metabolic dysfunction [73], demonstrating that circadian control of UPR aligns hepatic stress responses with protein-folding demands [73]. While this has been established in the liver, CPEB4’s known role in local translation in neurons suggests it may also couple circadian and stress responses in the brain. Altogether, these findings underscore the importance of circadian homeostasis in regulating cellular stress and its relevance to both metabolic and neuropsychiatric disorders.

### Oxidative stress and circadian rhythms

Circadian regulation of oxidative stress further reinforces the link between metabolism and psychiatric outcomes. Antioxidant enzymes, including catalase, superoxide dismutase, and glutathione peroxidase, are responsible for clearing harmful reactive oxygen species (ROS) and exhibit rhythmic expression patterns [74]. Disruptions in these rhythms, as seen in drosophila Period gene mutants, heighten ROS susceptibility and oxidative stress burden [75]. Likewise, sleep disturbance elevates oxidative stress in cortical rat neurons [76]. Additionally, mitochondrial dysfunction and oxidative stress contribute to the pathophysiology of BD 77, 78. Furthermore, BD patients with metabolic disorders such as T2D demonstrate accelerated cognitive decline [79], with oxidative stress being an independent contributor to the pathophysiology of metabolic disorders.

In toto, disruptions in sleep and circadian rhythms can trigger ER and oxidative stress, with each amplifying the other, thereby affecting both metabolic function and brain health. This intricate relationship underscores the critical role of circadian homeostasis in mitigating cellular stress and its broader implications for metabolic and neuropsychiatric disorders [80].

## Conclusion

The intricate interplay between circadian regulation, metabolism, and mental health underscores the need for integrative therapeutic strategies targeting circadian function. Circadian misalignment disrupts metabolic homeostasis, alters neurotransmitter signalling, and exacerbates mood instability, such that disruptions in one system reverberate across the others, creating a feedback loop that accelerates disease progression. In this review, we have synthesised emerging evidence, highlighting brain glucose, lipid signalling, and cellular stress as key mediators of shared molecular pathways. Targeting circadian regulation offers a unifying approach to simultaneously address metabolic and neuropsychiatric disorders. Large-scale, prospective clinical trials are needed to validate the long-term efficacy of circadian-based interventions. Future research should focus on elucidating these overlapping mechanisms and advancing precision medicine while leveraging existing therapies and lifestyle interventions to provide practical benefits. Integrating circadian biology into clinical practice may hold transformative potential for tackling the dual burdens of metabolic and psychiatric disease.

## Declaration of Competing Interest

The authors declare that they have no known competing financial interests or personal relationships that could have appeared to influence the work reported in this paper.

## Data Availability

No data were used for the research described in the article.
